# ChEMBL: towards direct deposition of bioassay data

**DOI:** 10.1093/nar/gky1075

**Published:** 2018-11-06

**Authors:** David Mendez, Anna Gaulton, A Patrícia Bento, Jon Chambers, Marleen De Veij, Eloy Félix, María Paula Magariños, Juan F Mosquera, Prudence Mutowo, Michał Nowotka, María Gordillo-Marañón, Fiona Hunter, Laura Junco, Grace Mugumbate, Milagros Rodriguez-Lopez, Francis Atkinson, Nicolas Bosc, Chris J Radoux, Aldo Segura-Cabrera, Anne Hersey, Andrew R Leach

**Affiliations:** 1 European Molecular Biology Laboratory, European Bioinformatics Institute, Wellcome Genome Campus, Hinxton, Cambridgeshire CB10 1SD, UK; 2 Open Targets, Wellcome Genome Campus, Hinxton, Cambridgeshire CB10 1SD, UK; 3 Institute of Cardiovascular Science, University College London, Gower Street, London WC1E 6BT, UK

## Abstract

ChEMBL is a large, open-access bioactivity database (https://www.ebi.ac.uk/chembl), previously described in the 2012, 2014 and 2017 Nucleic Acids Research Database Issues. In the last two years, several important improvements have been made to the database and are described here. These include more robust capture and representation of assay details; a new data deposition system, allowing updating of data sets and deposition of supplementary data; and a completely redesigned web interface, with enhanced search and filtering capabilities.

## INTRODUCTION

ChEMBL is a large, open-access drug discovery database that aims to capture Medicinal Chemistry data and knowledge across the pharmaceutical research and development process (1–3). Information about small molecules and their biological activity is extracted from the full text articles of several core Medicinal Chemistry journals and integrated with data on approved drugs and clinical development candidates, such as mechanism of action and therapeutic indications. Bioactivity data are also exchanged with other databases such as PubChem BioAssay (4) and BindingDB (5) allowing users to benefit from an even larger body of information. The resulting database has a wide variety of practical applications including the identification of chemical tools for a target of interest, assessment of compound selectivity, training machine learning models (e.g. for target prediction), assisting in generating drug repurposing hypotheses, assessing target tractability and integration into other drug discovery resources (6–12).

Extraction of compound, assay and bioactivity information from journal articles is performed manually by curators. This is a complex task that may involve, for example, the enumeration of specific chemical structures from an R-group table, reviewing cited references to establish assay details, or entering quantitative values and units from figure or table images. Even when performed by a domain expert, such processes may inevitably lead to inaccuracies such as incorrect compound connectivity or stereochemistry or unit transcription errors in recording measurements. The ChEMBL curation workflow therefore involves additional curation processes designed to identify, flag and correct errors. However, these can be time-consuming and it would be preferable if such issues could be avoided in the first place. One way to mitigate this problem would be to allow authors to deposit their bioactivity data into ChEMBL directly at the time of publication. This would have the advantage not only of reducing the error rate associated with subsequent data extraction (for example compound structures could be supplied in a machine-readable format rather than being redrawn), but it would also allow the capture of supplementary data and negative results, that may not be essential for inclusion in the publication but which may nevertheless enhance the structure-activity relationship (SAR) landscape for a target (13). In addition, data sets that are not associated with a journal publication may also be deposited, making them available to the community. Since its inception, ChEMBL has accepted data depositions and now contains >50 deposited data sets, particularly in the area of neglected disease drug discovery. However, as the number of depositors has grown, several problems have become apparent that we have sought to address. Extensions were required to the existing ChEMBL database schema to allow better capture of experimental details, and mechanisms were required to allow deposited data sets to be maintained and updated more easily. To this end, a range of new functionality has been incorporated into ChEMBL to facilitate data deposition, as described below. Further improvements to internal pipelines and release processes are also being implemented to allow for more frequent ChEMBL releases in the longer term (as would be required, for example, to allow for routine data deposition alongside journal publications).

Access to the data in ChEMBL is provided through a user interface, suite of web services and a number of download formats. The user interface previously had limited functionality in terms of searching and filtering data and has therefore undergone a complete redesign to provide users with the ability to address a broader range of use-cases and a better user experience. These improvements are described in detail in the following sections.

## ChEMBL DATA CONTENT

A large proportion of the activity data in ChEMBL is currently extracted manually from full text articles in seven Medicinal Chemistry journals: MedChemComm, Journal of Medicinal Chemistry, ACS Medicinal Chemistry Letters, European Journal of Medicinal Chemistry, Bioorganic & Medicinal Chemistry, Bioorganic & Medicinal Chemistry Letters, Journal of Natural Products. For these journals, every article in each new issue is screened for the presence of quantitative small molecule (or peptide) bioactivity data. Data are also sometimes extracted from other journals and articles, when relevant to an area of particular interest (see Table 1). For example, a large set of crop protection data was added to the database in previous years (14), and more recently, data from the *Journal of Drug Metabolism and Disposition* have been included (3).

**Table 1.** Top 15 journals covered by ChEMBL (release 24), according to numbers of articles extracted

Journal	Number of documents
Bioorg. Med. Chem. Lett.	21 197
J. Med. Chem.	21 032
Bioorg. Med. Chem.	6996
J. Nat. Prod.	6701
Eur. J. Med. Chem.	5514
Antimicrob. Agents Chemother.	2121
ACS Med. Chem. Lett.	1378
Med. Chem. Res.	1309
MedChemComm	892
J. Agric. Food Chem.	422
Drug Metab. Dispos.	272
J. Pesticide Sci.	245
Nat. Chem. Biol.	170
Crop Protection	129
Pest. Manag. Sci.	126
Others	570

From each article, details of compounds tested, assays performed, endpoints measured and relevant target information, are extracted. Compound structures are drawn in full (including any salt present) and saved in V2000 Mol file format. A brief description of the assay is abstracted; this typically includes the type of assay being performed; any cell-line, tissue or organism used; the property being assessed and the target (where applicable). Biological activity data is recorded; this includes (but is not limited to) binding measurements, efficacy in functional assays, pharmacokinetic data and toxicity endpoints. Each assay is classified into one of the following categories: B (binding assay), F (functional assay), A (ADME assay), T (toxicity assay), P (physicochemical assay) or U (unclassified assay). Where the assay describes the interaction with a molecular target, this target is also recorded in the form of a UniProt (15) accession, or list of accessions. Quantitative and qualitative activity measurements are extracted in the form reported in the publication, with their respective activity types, units and qualifiers.

Following initial data extraction, an extensive data curation and standardization process is applied before incorporating the information into ChEMBL. This process has been described in detail previously (14,16); in brief, compound structures are standardized, salt-stripped and assigned identifiers based on Standard InChI (17); assay descriptions are mapped to controlled vocabularies such as the Cell Line Ontology (18), Uberon (19) and BioAssay Ontology (20); activity measurements and units are converted to a standard form; and multi-protein targets are created where required. This same curation process is also applied to other data sources within ChEMBL such as data extracted from patents, deposited data sets (often in collaboration with the depositor) and data from PubChem BioAssay and BindingDB.

This detailed standardization process allows users to more easily retrieve, compare and analyse data of interest. For example, via the user interface, users can view a parent compound and see bioactivity data measured against all salt forms of the compound (though the data are still stored against the relevant salt) rather than having to query for each salt form individually. A user wishing to filter bioactivity data and only retrieve compounds more active than a particular activity threshold can safely assume nM units for commonly used dose-response measurements such as IC50, Ki or EC50. Relationships between targets are also shown, so that a user interested in the GABA-A receptor alpha-1 subunit, for example, can see that data are available for a number of different protein complexes containing this protein.

### Current data content

Release 24 of the ChEMBL database contains bioactivity information extracted from >67 000 publications and patents, alongside deposited data sets and data exchanged with other databases such as PubChem BioAssay and BindingDB (a full list of sources with the number of data points from each can always be found in the latest release notes at: ftp://ftp.ebi.ac.uk/pub/databases/chembl/ChEMBLdb/latest). In total, ChEMBL now contains a total of >15 million bioactivity measurements for 1.8 million distinct compounds. Assays are annotated with >1600 distinct cell lines, 500 tissues/organs and 3600 organisms. Approximately two thirds of these assays measure activity of compounds against human, mouse or rat targets (or *in vivo* endpoints), but there are also significant numbers of assays studying other model organisms or pathogenic species. For example, ChEMBL currently contains around 21 000 assays against *Staphylococcus aureus* and 10 000 assays against *Human immunodeficiency virus 1*, making it a rich source of information for anti-infective drug discovery efforts. The assays and compounds in ChEMBL target >8200 proteins, including 3569 human proteins.

### New data sources

Since the 2017 description of the database, a number of new data sources have been incorporated into ChEMBL, as outlined below.

#### Patent bioactivity data

In addition to data from the peer-reviewed literature, ChEMBL now incorporates some data extracted from patent documents. This data extraction effort is focused on patents containing compounds and bioactivity data for targets that are not currently well represented in ChEMBL (and in particular, those of interest to the NIH Illuminating the Druggable Genome project: https://commonfund.nih.gov/IDG/understudiedproteins (7)). To date, 74 050 activity measurements have been extracted from 241 patents, and these are available in ChEMBL as source 38. Additional, focused sets may also be included in future releases, where these add value to the existing ChEMBL data. A larger set of bioactivity data extracted from granted US patents by BindingDB (described previously) is also available in ChEMBL as source 37 (3).

#### Curated drug pharmacokinetic data

Knowledge of the pharmacokinetic properties of drugs is critical in understanding their safety and efficacy profiles, yet such data are often not readily available in a structured form. In order to begin to address this, pharmacokinetic measurements for 85 drugs have been extracted from reference books (21) and drug prescribing information (https://dailymed.nlm.nih.gov/dailymed/) and incorporated into ChEMBL as source 39.

#### CO-ADD antimicrobial screening data

The Community for Open Antimicrobial Drug Discovery is a not-for-profit initiative led by the University of Queensland (http://www.co-add.org). The goal of the project is to screen compounds for antimicrobial activity on behalf of academic research groups. Screening results from the CO-ADD project are being deposited in the ChEMBL database. The first such data set reports the results of screening Open Source Malaria (http://opensourcemalaria.org) compounds in the CO-ADD assays. This data set consists of 20 compounds tested in up to 15 assays, resulting in a total of 180 bioactivity data points. The data set can be located with the DOI: 10.6019/CHEMBL3832881, source 40. Further CO-ADD data sets will be deposited in ChEMBL in the near future.

#### K4DD project

K4DD is an Innovative Medicines Initiative-funded project with the goal of driving the greater use of drug-target binding kinetics in the drug discovery decision-making process (https://www.k4dd.eu/home/) (22). Following the completion of the project in 2017, kinetic data generated by participants was deposited in ChEMBL (DOI: 10.6019/CHEMBL3885741, source 30). The data set consists of 2064 measurements (including *k*_on_ and *k*_off_ values) for 273 compounds in 48 assays.

#### MMV pathogen box

The MMV Pathogen Box (https://www.pathogenbox.org) is a set of 400 diverse, drug-like compounds with activities in a range of neglected diseases including Tuberculosis, Malaria, Chagas disease, Leishmaniasis, Human African Trypanosomiasis, Cryptosporidiosis, Lymphatic Filariasis, Onchocerciasis, Schistosomiasis, Dengue, Chikungunya and Toxoplasmosis. The compound collection is deposited in ChEMBL, together with an initial set of bioactivity measurements under the DOI: 10.6019/CHEMBL3832761, source 34. A second set of Pathogen Box bioactivity data has also been deposited in ChEMBL. This data set describes the screening of the Pathogen Box against human ferrochelatase and porphobilinogen deaminase by the Australian National University and is deposited with DOI: 10.6019/CHEMBL3987221.

### Drugs and clinical candidates

The ChEMBL team continue to curate therapeutic targets and indications for clinical candidates and approved drugs. Compounds in clinical development are identified from two main sources – United States Adopted Name (USAN) applications (https://www.ama-assn.org/about/usan-council) and the ClinicalTrials.gov database (https://clinicaltrials.gov). Approved drugs are identified primarily from the FDA Orange Book database (https://www.accessdata.fda.gov/scripts/cder/ob/) and the annual list of FDA New Drug Approvals (https://www.fda.gov/Drugs/DevelopmentApprovalProcess/DrugInnovation/default.htm), but information is also extracted from the British National Formulary (https://bnf.nice.org.uk) and the ATC classification (https://www.whocc.no/atc_ddd_index/). Indications for clinical candidates are obtained from ClinicalTrials.gov (https://clinicaltrials.gov) and are mapped to Medical Subject Headings (MeSH; https://www.nlm.nih.gov/mesh/) and Experimental Factor Ontology (EFO (23)) disease identifiers through a combination of manual and automated approaches. Indications for approved drugs are obtained from DailyMed (https://dailymed.nlm.nih.gov/dailymed/) package labels and the ATC classification. Therapeutic targets for both approved drugs and clinical candidates are manually assigned using reference sources such as scientific literature, drug package labels and company pipeline information. In each case, references are captured indicating the original source of the information. Considering parent compounds (rather than individual salts), the current release of ChEMBL contains 10,492 compounds with USAN applications, 5354 compounds that are recorded to have reached at least phase I clinical trials, and 2715 approved drugs. Of these clinical-stage compounds, 4875 have one or more indications assigned and 3907 have a curated mechanism of action. Information about drugs that have been withdrawn from the market is also included in ChEMBL, and those drugs withdrawn for toxicity reasons are categorized according to the type of toxicity shown (e.g. hepatotoxicity, cardiotoxicity, neurotoxicity).

### Calculated properties

Prior to release 24 of ChEMBL, compound physiochemical properties were calculated using Pipeline Pilot (version 8.5, Accelrys Inc. 2012) and ACD/Labs Physchem software (version 12.01, Advanced Chemistry Development Inc. 2010). From ChEMBL_24 onwards, the majority of the properties (MW_FREEBASE, ALOGP, HBA, HBD, PSA, RTB, QED_WEIGHTED, FULL_MWT, AROMATIC_RINGS, HEAVY_ATOMS, MW_MONOISOTOPIC, FULL_MOLFORMULA, HBA_LIPINSKI and HBD_LIPINSKI) are now being calculated using RDKit (https://www.rdkit.org, 2018), with ACD_MOST_APKA, ACD_MOST_BPKA, ACD_LOGP, ACD_LOGD and MOLECULAR_SPECIES still calculated with ACD/Labs software. As a result, the values of some properties may have changed slightly from previous releases. More details on these properties and their calculation are available at: https://chembl.gitbook.io/chembl-interface-documentation/frequently-asked-questions/chembl-data-questions#how-is-the-logp-calculated.

## DATA DEPOSITION

As indicated above, ChEMBL has accepted direct data depositions since its inception, and release 24 of the database contains more than 50 such data sets from collaborators including MMV, DNDi, GSK and AstraZeneca. Each deposited data set is assigned a Digital Object Identifier (DOI), which resolves to the appropriate ChEMBL page, making it possible to permanently reference the data set. For example, the set of experimental in vitro DMPK and physicochemical data deposited by AstraZeneca has been assigned the DOI: 10.6019/CHEMBL3301361. This resolves to the Document Report Card for the data set, allowing users to directly and easily access the 11 687 activity measurements and 5799 compounds deposited. However, since ChEMBL was originally designed to capture data extracted from peer-reviewed literature, some aspects of the database schema, loader and release process were not optimized for large-scale data deposition. Improvements that allow richer capture of assay protocol information, updates of previous data depositions and better representation of provenance are described below.

### Assay protocol information

Assay protocol information in ChEMBL was historically captured in the form of a brief assay description, together with a few key fields such as assay type, cell line, tissue and target information. While such a summary might not always provide everything that a user might want to know about the experiment performed, it was nevertheless straightforward, where required, to link back to the original journal article and obtain more fine-grained details such as the detection method, instrument or type of media used. In the case of deposited data sets, however, an online or published assay protocol may not always exist. It has therefore become increasingly important to allow the capture of more detailed assay information within ChEMBL. Given the wide range, variability and complexity of drug discovery and development assays, this is a challenging database and schema design task.

#### Assay parameters

An ASSAY_PARAMETERS table was introduced in previous releases of ChEMBL to allow the capture of any user-defined parameters as simple key-value pairs (for example, ‘DOSE: 5’, ‘DOSE_UNITS: mg.kg-1’). The format of this table has now been extended to capture parameter information in a more structured form and to allow standardization. The new table captures the parameter TYPE, RELATION, VALUE (or TEXT_VALUE) and UNITS as submitted or extracted, with a second set of columns (STANDARD_TYPE, STANDARD_RELATION, STANDARD_VALUE, STANDARD_UNITS and STANDARD_TEXT_VALUE) that allow for the parameter types, values and units to be standardized as per the process for the main activities table (16). Examples of the types of parameters that could be captured in this table include temperature, pH, media type, detection method or compound concentration. Such parameters should apply to the assay as a whole (i.e. be relevant to all compounds tested and measurements recorded).

#### Activity properties

While the ASSAY_PARAMETERS table captures variables that relate to the experiment as a whole, it is sometimes necessary to capture variables that relate only to certain activity measurements. For example, in an *in vivo* assay intended to measure drug pharmacokinetic, safety pharmacology or efficacy information, different compounds may be administered at different doses or by different routes. Measurements may also be taken at different time points or in different tissues. In previous releases of ChEMBL, it was necessary to register separate assays for each compound, administration route, time point or tissue in order to capture these details. This resulted in many assays being artificially divided (particularly in the large Open TG-GATEs and DrugMatrix data sets), presenting some difficulties for users in retrieving, integrating and interpreting the data. In order to overcome these problems, the ACTIVITY_PROPERTIES table has been introduced. This takes more or less the same format as described above for the ASSAY_PARAMETERS table, but the properties stored in this table are linked to individual activity measurements rather than to an assay. In addition to storing independent variables, the ACTIVITY_PROPERTIES table can also be used to record properties or dependent variables that are considered important when interpreting the measurements recorded in the main ACTIVITIES table, e.g. the Hill slope or maximum effect for an IC50 determination. In such cases, a RESULT_FLAG is set to 1 (rather than 0) to distinguish these measured properties from independent variables/parameters such as the compound dose, administration route or time point.

#### Supplementary Data

In many assays, several measurements are recorded for each compound—for example, multiple different endpoints may be measured in parallel and then converted into a single combined result, or alternatively a summary measurement may be calculated from multiple individual data points or replicates. In many cases, such summary measurements are sufficient for ChEMBL users. However, for those cases where additional information would be valuable in properly interpreting the experimental results, a mechanism has been implemented to allow these to be captured. A new ACTIVITY_SUPP table allows supplementary measurements to be linked to measurements in the main ACTIVITIES table. For example, in an *in vivo* pathology experiment, the ACTIVITIES table may hold data summarized across a group of animals, with individual animal-level data optionally recorded in the ACTIVITY_SUPP table. Similarly, where necessary, the individual inhibition measurements (points on the curve) contributing to an IC50 determination could be stored in the ACTIVITY_SUPP table and linked to the IC_50_ value in the ACTIVITIES table.

### Data deposition format

In order to deposit data in ChEMBL, submitters should include their data in a set of tab-separated files providing details of the references associated with the data (either a description of the data set or publication), the compounds tested (with a separate SD file for the compound structures), the assays performed, the activity measurements and, optionally, any assay parameters or activity properties. The format of these files is described in detail in documentation provided on the ChEMBL-NTD FTP site (ftp://ftp.ebi.ac.uk/pub/databases/chembl/ChEMBLNTD/Deposition_Documentation/) and a template with examples is also provided (ftp://ftp.ebi.ac.uk/pub/databases/chembl/ChEMBLNTD/ChEMBL_Deposition_Template.tar.gz). Systems have been put in place to check and validate submitted files; for example, to check that all required fields are present and have the correct data types, but also to check compound structures for any problems before data are loaded to ChEMBL. Work is also underway to develop a web-based submission tool that will allow users to submit and validate their own deposition files directly in future.

A change to the previous data deposition system is the introduction of depositor-defined IDs for compounds, assays and references/documents. These fields are referred to as CIDX (for compounds), AIDX (for assays) and RIDX (for references) and are, as the name implies, defined by the depositor. The identifiers used should ideally be meaningful to the data provider, as they need to remain stable between data depositions (i.e. if a subsequent data set contains any of the same compounds, assays or references as an earlier deposition, they must be referred to by the same identifiers). This system allows for existing data sets to be updated (for example to correct an error), for further data to be added against existing sets of compounds or assays, and for activity data to be uploaded against another depositor's compounds or assays (for example, a group screening the MMV Pathogen Box compound set could upload their own assay and activity data and refer to the CIDXs of the Pathogen Box compound set).

It should be noted that PubChem BioAssay also accepts deposited bioactivity data. Since ChEMBL and PubChem exchange data, any data sets deposited in ChEMBL will also be incorporated into PubChem. Similarly, data deposited into PubChem will be loaded into ChEMBL if it meets the criteria for inclusion (e.g. confirmatory assays: https://chembl.gitbook.io/chembl-interface-documentation/frequently-asked-questions/chembl-data-questions#which-sub-set-of-the-pubchem-bioassay-data-has-been-integrated-into-chembl). Therefore, the decision as to where to deposit data is at the discretion of the depositor and will be influenced by a number of factors such as the type of data, experience from previous depositions or personal preference. Potential depositors wishing to check the suitability of their data for inclusion in ChEMBL or requiring help with the deposition format are advised to contact the ChEMBL Team, who will be happy to provide guidance and assistance.

### ChEMBL data model

The introduction of activity properties and the changes made to the data deposition system result in some implicit changes to the ChEMBL data model. Previously it was the case that both compounds and assays (and therefore also activities) in a dataset referred to the same document and had the same source (e.g., depositor). When querying ChEMBL, the src_id or doc_id could be taken from either the COMPOUND_RECORDS table or ASSAYS table and be assumed to be the same in either case. This is no longer necessarily the case; it is now possible for activity measurements to have a different source and document from the compounds and assays that they are associated with. For this reason, a src_id field has been added to both the DOCS and ACTIVITIES tables.

The introduction of the ACTIVITY_PROPERTIES table means that certain types of assays no longer need to be subdivided into separate ChEMBL assays in order to record parameters such as compound dose or time point information. Therefore, users should be aware that in future, some assays may have multiple activity measurements recorded for the same compound and assay, differing in properties such as the time point of the measurement. In order to take advantage of the changes in data model and schema, several existing data sets have been reformatted since previous releases. In particular, the Open TG-GATEs and DrugMatrix *in vivo* data sets have been extensively revised and now consist of just two assays each (corresponding to single and daily repeat regimens), rather than the hundreds of thousands of separate assays that were previously required to represent the different compound doses, administration routes, time points and tissues assessed (now captured in the ACTIVITY_PROPERTIES table).

## DATA ACCESS

### Web interface

The legacy ChEMBL web interface enabled users to search for compounds or targets of interest and retrieve bioactivity data. However, several important capabilities, such as viewing compound selectivity data or filtering data on desired properties, were difficult to perform and often required users to download data (e.g. as an Excel table) and perform these relatively straightforward tasks independently. The ChEMBL interface has therefore been completely redesigned to support a much wider range of functionality. Here, we describe the key design and user features of the new interface, which has recently been made available to the user community (https://www.ebi.ac.uk/chembl/beta/) and will replace the legacy interface in early 2019. The new web interface has been built entirely using the ChEMBL web services and is also designed to incorporate many of the features and characteristics present in contemporary websites.

#### Improved free-text searching

**Figure 1.**
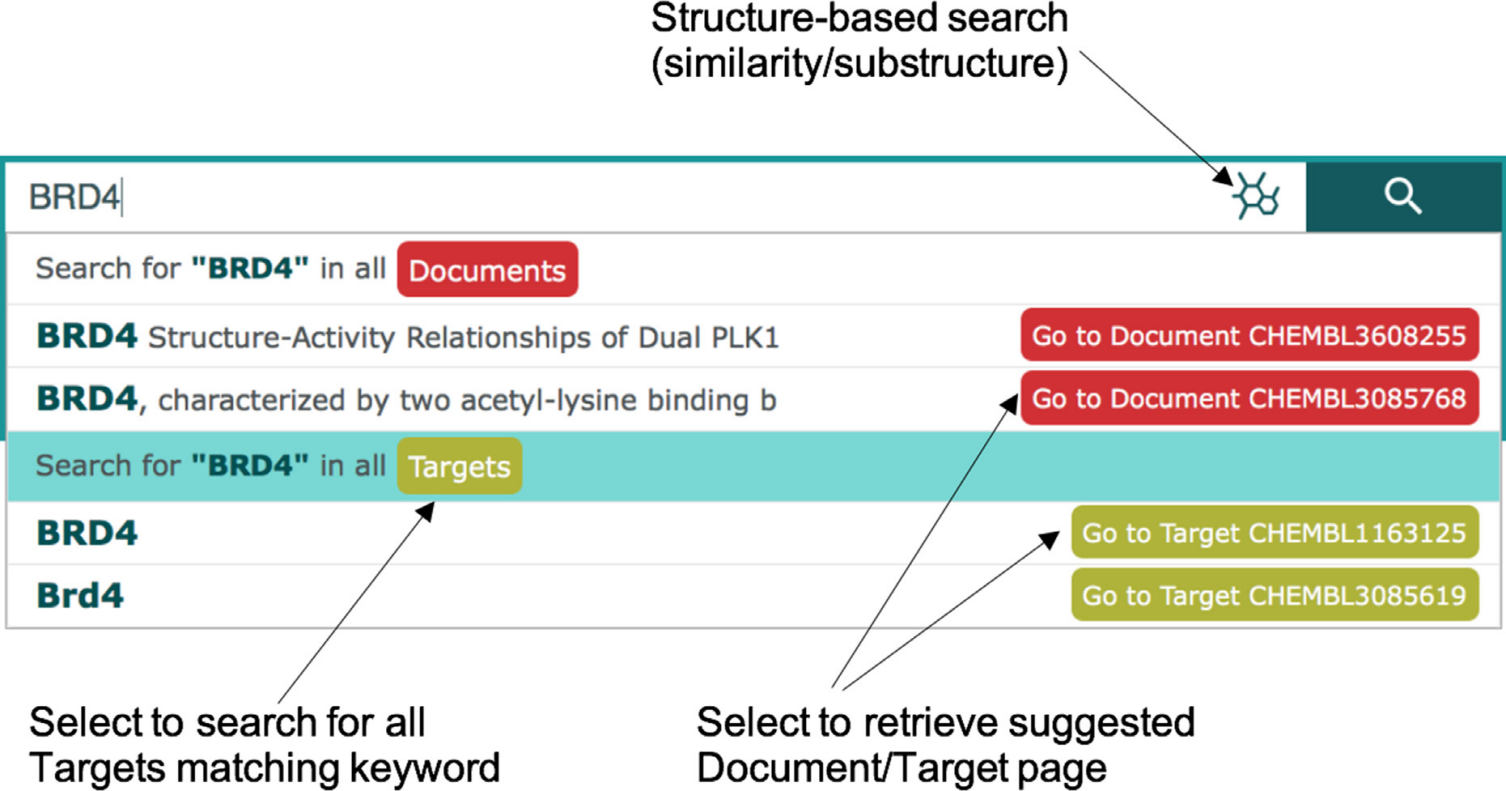
Autocomplete function of the search bar in the new web interface. Users can retrieve a list of entities matching a selected keyword (e.g. all Targets matching ‘BRD4’) or go directly to a Report Card for a selected entity (e.g. human BRD4: CHEMBL1163125).

In the legacy interface it was necessary to specify a search entity (Compound, Target, Assay, Document, Cell Line or Tissue) in order to retrieve the results. The new interface automatically searches among all the main entities in ChEMBL to identify potential matches. The search bar also provides an *autocomplete* functionality, showing suggestions as the term is written. This is especially useful when writing technical terms or to guide the user to identify the correct search term (Figure 1). Free-text search in the new ChEMBL interface uses a custom DSL (Domain Specific Language) based on the Query String DSL (https://www.elastic.co/guide/en/elasticsearch/reference/current/query-dsl-query-string-query.html) of Elasticsearch (https://www.elastic.co/products/elasticsearch). This allows users to define multiple types including SMILES, InChI and InChI Key and get direct results. The goal is to recognize different chemical and biological formats and provide accurate results accordingly. It is also possible to specify a list of search terms (e.g. ChEMBL IDs), separated by spaces, in the search bar and retrieve the relevant entities.

#### Interactive filtering

**Figure 2.**
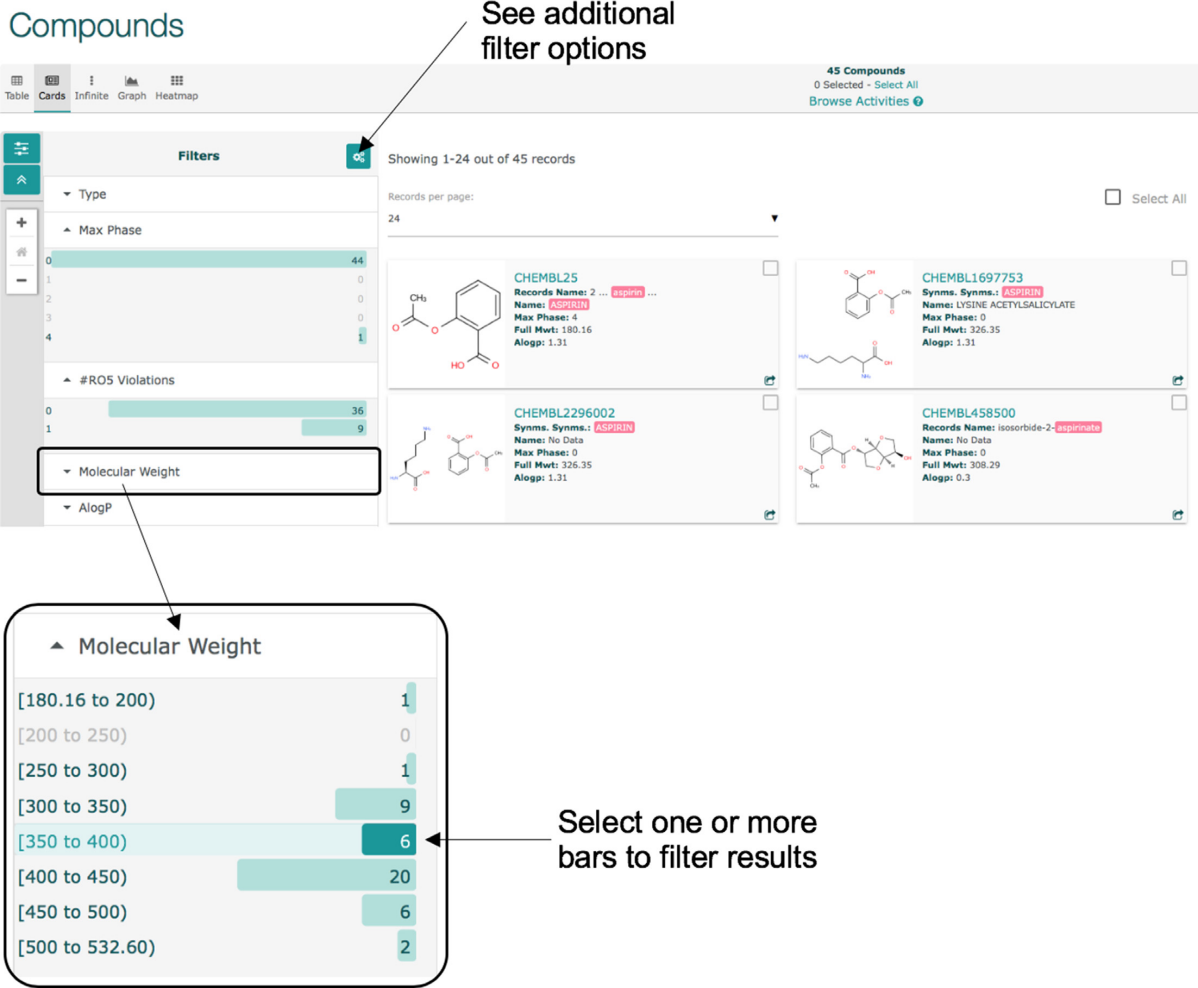
Filtering search results on the new ChEMBL interface. A default set of filters are displayed for each entity type (e.g. compounds shown here), but further filters can be added using the settings icon at the top right of the ‘Filters’ panel. Multiple filters from the same or different categories can be selected by clicking on the bars.

The new interface provides several preconfigured filters that can be applied when browsing a hitlist. For example, for the set of compounds obtained using the search term ‘Aspirin’ (see https://www.ebi.ac.uk/chembl/beta/g/#search_results/all/query=Aspirin), the interface will show interactive filters that include the molecule type, max phase, number of rule-of-five violations and molecular weight, as shown in Figure 2. The filters have two main functions: to show the distribution of the data set with regard to a specific property, and to allow the user to browse a subset of the original data in a given range for the filter property. Figure 2 provides an example of an interactive filter for the compounds found after searching for the term ‘Aspirin’, showing a histogram of the molecular weight distribution of the 45 compounds obtained by the query. Clicking on the section with label ‘[350 to 400]’ will display just the 6 compounds with a molecular weight between 350 and 400. Further filter options can be added by selecting the filter settings icon above the current filters.

#### Data visualizations

**Figure 3.**
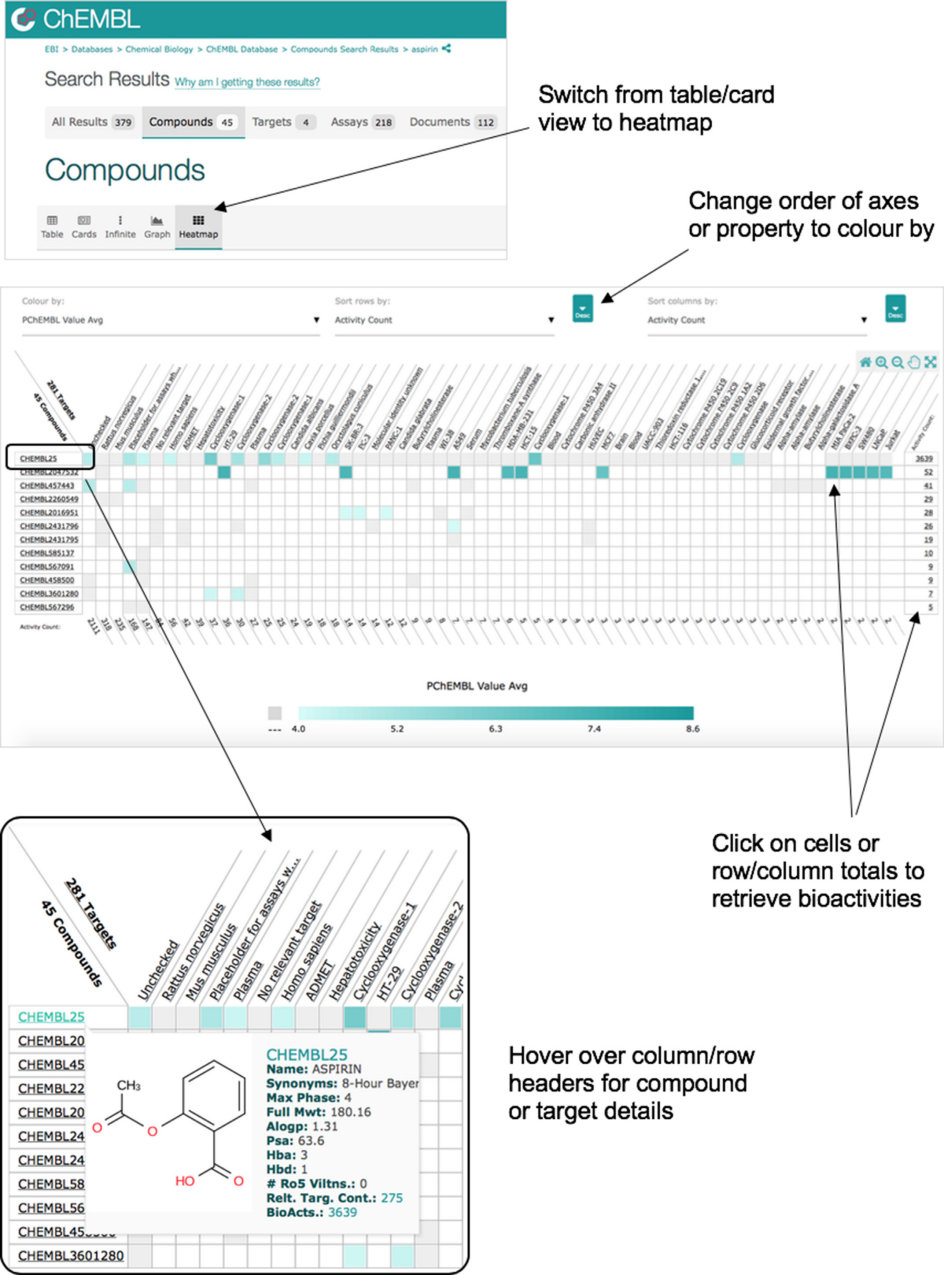
Heatmap visualization. The Heatmap view can be invoked from a set of compound/target search results by selecting the ‘Heatmap’ icon from the top-left menu bar. This will produce a heatmap showing the selected compounds or targets plotted against the targets or compounds with which they have been shown (or tested) to interact. Clicking on a cell will allow retrieval of the bioactivity data supporting that compound/target interaction.

To help users to better understand the data inside ChEMBL, several data visualizations have been created. These include the ‘Bioactivity Heatmap’, which shows the bioactivity relationships between compounds and targets. For any subset of compounds or targets (where each contains fewer than 1024 items), a heatmap can be generated by clicking the ‘Heatmap’ tab (see Figure 3). Each cell in the heatmap represents the interaction between a compound and a target. Where there is data in ChEMBL for this interaction pair then the cell is coloured to represent all activity data relating the two items (an example of the heatmap can be seen by visiting https://www.ebi.ac.uk/chembl/beta/g/tiny/uxXb0y9oAGeMqaF5Ah8pmQ==). Hovering on the row or column headers displays a mini report card of the corresponding compound or target. The cells can be coloured to represent different quantities such as the activity count or the average pChEMBL value. Clicking on a cell retrieves the bioactivity data for a particular compound and target.

**Figure 4.**
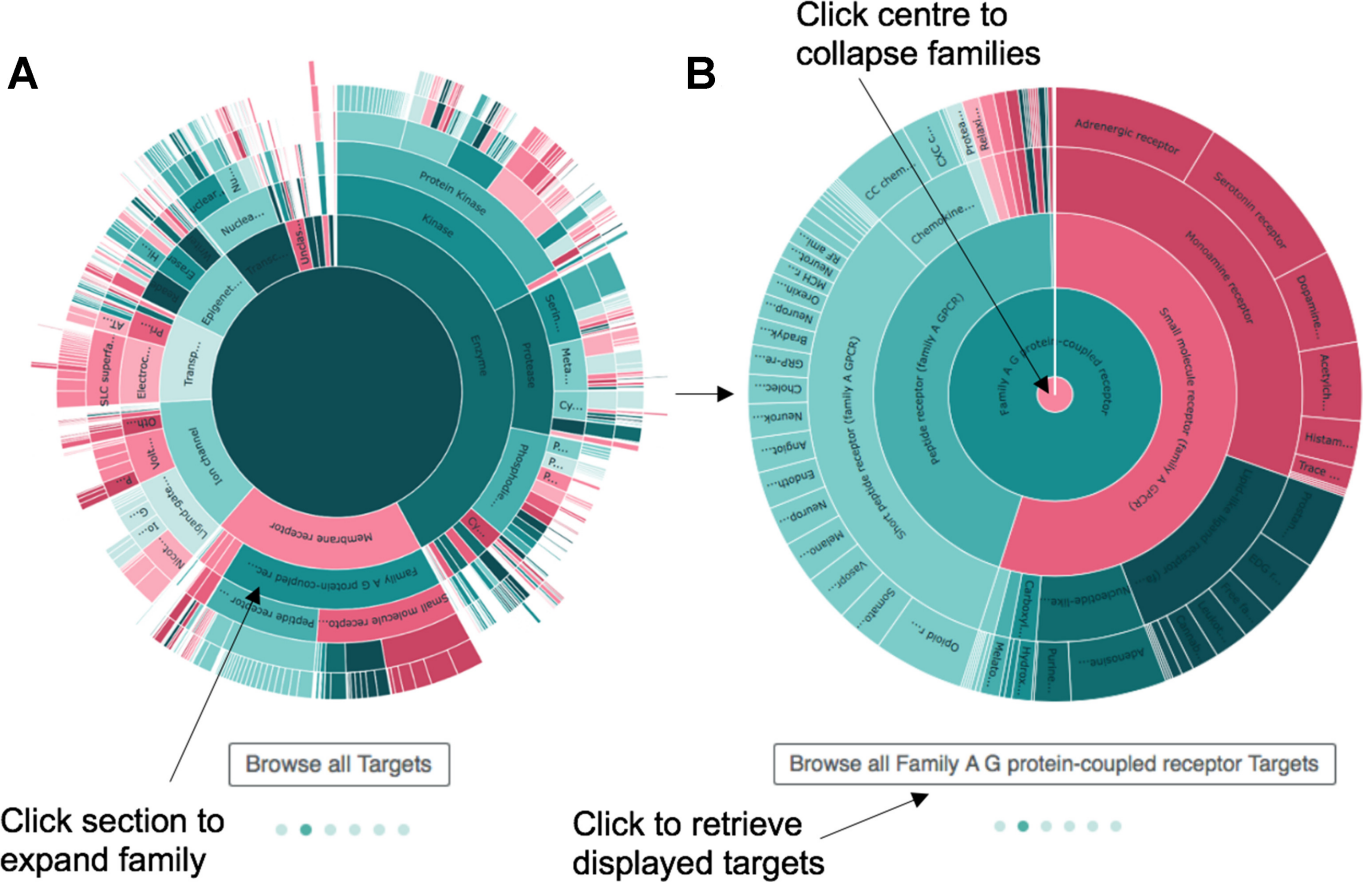
Sunburst visualization showing the ChEMBL protein family classification. (**A**) The classification is arranged in concentric circles with major families in the centre and smaller subfamilies around the edge. (**B**) To zoom in or expand a family, the user can click the relevant segment in the visualization and the resulting set of targets can be retrieved using a button below the plot.

Other visualizations have also been created and can be viewed in the main page of the new interface or in the visualizations page (https://www.ebi.ac.uk/chembl/beta/visualise/). Two visualizations in particular have been created to help users browse different ChEMBL data hierarchies. An interactive *sunburst* has been created to explore the protein target classification (see Figure 4A). Protein families can be expanded by clicking a section of the plot (Figure 4B) and the corresponding targets can be retrieved using the button below the chart. A *circle chart* has also been created to explore the organism taxonomy classification of the targets.

#### Browsing related activity data

**Figure 5.**
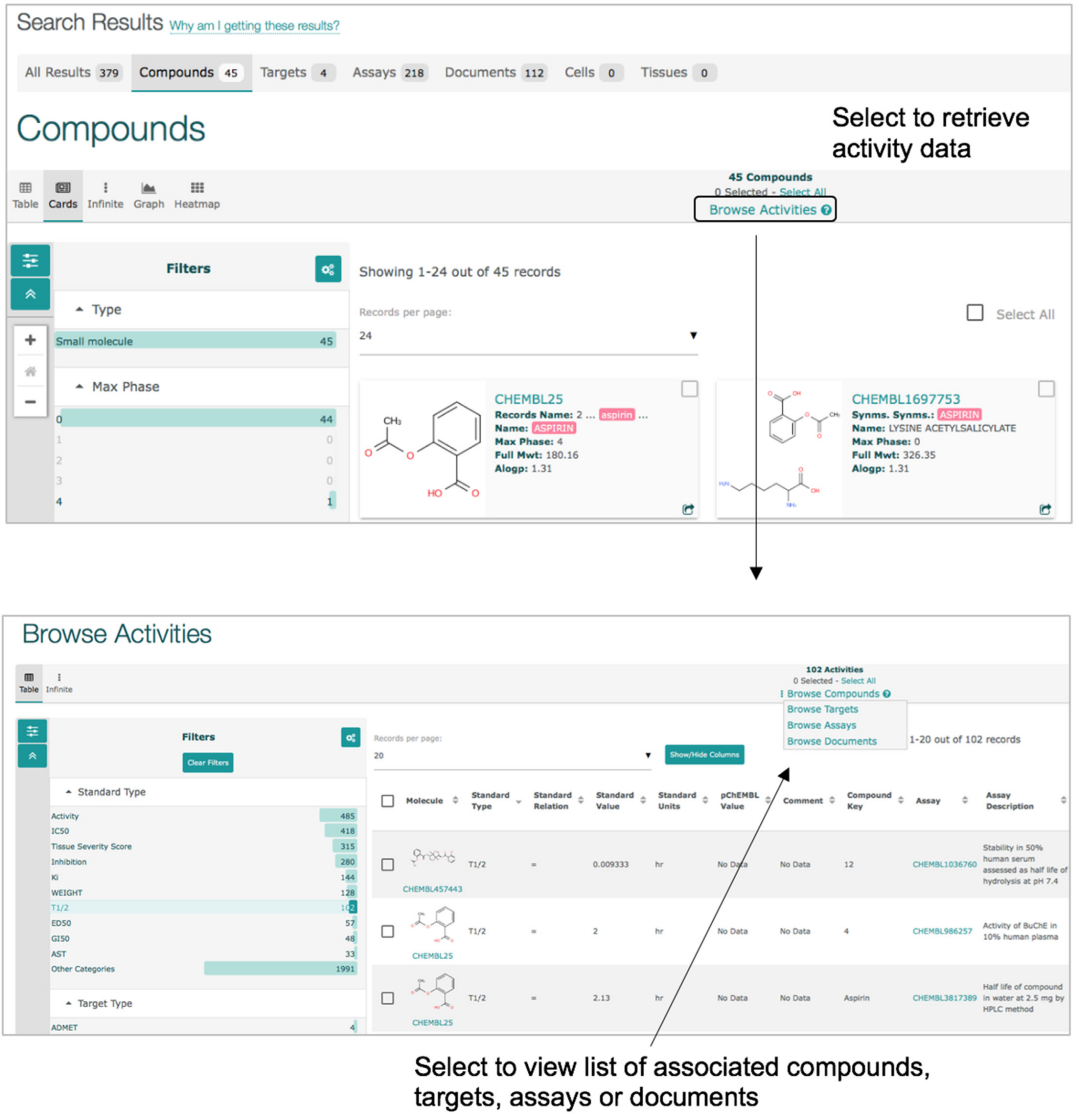
Retrieval of bioactivity data from search results. The ‘Browse Activities’ link above the table of search results retrieves the set of activity measurements associated with the selected entities. From this view the user can further retrieve the distinct list of compounds/targets/assays/documents, again by selecting the link above the table.

In the new interface it is possible to access related activity data much more easily than previously. To continue with our ‘Aspirin’ example, a ‘Browse Activities’ button will appear in the top menu (Figure 5) which, when clicked, provides the activities related to those 45 compounds. Similarly, when browsing activities, the user can jump to other related entities using the menu above the results table.

#### Saving the state in the URLs

One of the goals of the new interface is to allow users to share the results of their work with others. This is achieved by saving the state in the URLs. As is common practice, this is done via shortened URLs which can then be easily shared by email, chat, and social media. After performing a particular query and/or filtering results, a shortened URL can be obtained by clicking the share icon in the breadcrumbs section at the top of the page (see https://chembl.gitbook.io/chembl-interface-documentation/#descriptive-urls for more information). The following are examples of the link shortening and state saving:

- https://www.ebi.ac.uk/chembl/beta/g/tiny/uxXb0y9oAGeMqaF5Ah8pmQ== (Compound versus Targets heatmap in full screen)
- https://www.ebi.ac.uk/chembl/beta/g/tiny/GvKCBuljiVr_s1XL8m6Duw== (All the targets related to the compound CHEMBL25)
- https://www.ebi.ac.uk/chembl/beta/g/tiny/gR_Sn2Ox0WQ1D3BLqjvCIQ== (All the compounds related to the target CHEMBL5016 that are in phase 4.

Using these and other capabilities, users can now undertake much more sophisticated filtering and visualization of their ChEMBL data. This includes the identification of particular subsets of interest, for example all assays carried out on protein kinases, binding measurements for G protein-coupled receptors, or ADME assays carried out in rat, replacing much of the functionality previously available in the (now deprecated) SARfari tools. There will nevertheless always be a limit to the capabilities that can be easily provided via a web interface. For more in-depth and specific use cases, users can use the ChEMBL Web Services, or download the entire database.

Other help and information about using the new user interface can be found on the documentation pages: https://chembl.gitbook.io/chembl-interface-documentation/.

### Web services

For users preferring to access ChEMBL programmatically, a comprehensive set of RESTful web services are provided (24,25), allowing retrieval of ChEMBL data in XML, JSON and YAML formats. Interactive documentation is available, allowing users to test services and view the output (https://www.ebi.ac.uk/chembl/api/data/docs) and documentation and usage examples can be found on the web services home page: https://www.ebi.ac.uk/chembl/ws. A Python web service client library is also available: https://github.com/chembl/chembl_webresource_client. The ChEMBL web services are open source, available from the ChEMBL GitHub repository (https://github.com/chembl/) and are licensed under an Apache 2 license.

### Downloads

Some users may prefer to download the database and query it locally (e.g. for use in data mining applications or to integrate with internal data), rather than via the user interface or web services. Each release of the database is available from the ChEMBL ftp site (ftp://ftp.ebi.ac.uk/pub/databases/chembl/ChEMBLdb/latest/) in a variety of formats including: Oracle, MySQL, PostgreSQL, SQLite, RDF (26), an SD file of compound structures and a FASTA file of the target sequences.

## SUMMARY

The ChEMBL database is a widely used drug discovery resource, with a global user base in academia, industry and charitable organisations. In the almost 10 years since it was introduced as an open access resource, it has provided the impetus for new discoveries, the creation of new spin-out companies and the validation of computational tools. The current emphasis on Artificial Intelligence approaches and applications provides yet further evidence of the value that high-quality, curated resources such as ChEMBL offer. At the same time, it is important that such resources ‘move with the times’, not only continuing to deliver quality content but also providing users with the ability to more easily interact with the data and to facilitate integration with other data types. In this paper we have described in detail two aspects of the current ChEMBL system that we believe will offer both expert and non-expert users access to greater capabilities in the broad area of drug discovery data and informatics.

## DATA AVAILABILITY

The ChEMBL database is made available under a Creative Commons Attribution-ShareAlike 3.0 Unported license (http://creativecommons.org/licenses/by-sa/3.0).
